# Development and Preclinical Evaluation of [^68^Ga]BMSH as a New Potent Positron Emission Tomography Tracer for Imaging Programmed Death-Ligand 1 Expression

**DOI:** 10.3390/ph16101487

**Published:** 2023-10-19

**Authors:** Yong Huang, Chengze Li, Zhongjing Li, Qiong Wang, Size Huang, Qi Liu, Ying Liang

**Affiliations:** 1 Department of Nuclear Medicine, National Cancer Center, National Clinical Research Center for Cancer, Cancer Hospital & Shenzhen Hospital, Chinese Academy of Medical Sciences and Peking Union Medical College, Shenzhen 518116, China; 13260455651@163.com (Y.H.); lichengze1915@163.com (C.L.); lizhongjing321@163.com (Z.L.); 18730288973@163.com (Q.W.); my1005311039@163.com (S.H.); 2 International Cancer Center, Shenzhen University School of Medicine, Shenzhen University, Shenzhen 518057, China; 3 Institute of Biomedical Engineering, Shenzhen Graduate School, Peking University, Shenzhen 518055, China

**Keywords:** [^68^Ga]BMSH, immunotherapy, programmed death-ligand 1, positron emission-computed tomography

## Abstract

Immunotherapy targeting the programmed death-ligand 1 (PD-L1)/programmed cell death protein 1 (PD-1) pathway has shown remarkable efficacy against various cancers, but the overall response rate (ORR) is still low. PD-L1 expression in tumors may predict treatment response to immunotherapy. Indeed, ongoing clinical studies utilize a few PD-L1 radiotracers to assess PD-L1 expression as a predictive biomarker for immunotherapy. Here, we present a novel positron emission tomography (PET) radiotracer called [^68^Ga]BMSH, which is derived from a small molecule inhibitor specifically targeting the binding site of PD-L1. The inhibitor was modified to optimize its in vivo pharmacokinetic properties and enable chelation of ^68^Ga. In vitro evaluation revealed [^68^Ga]BMSH possessed a strong binding affinity, high specificity, and rapid internalization in PD-L1 overexpressing cells. Biodistribution studies showed that PD-L1 overexpressing tumors had an uptake of [^68^Ga]BMSH at 4.22 ± 0.65%ID/g in mice, while the number was 2.23 ± 0.41%ID/g in PD-L1 low-expressing tumors. Micro-PET/CT imaging of tumor-bearing mice further confirmed that, compared to [^18^F]FDG, [^68^Ga]BMSH can specifically identify tumors with varying levels of PD-L1 expression. Our findings suggest that the [^68^Ga]BMSH is a PD-L1 radioligand with ideal imaging properties, and its further application in the clinical screening of PD-L1 overexpressing tumors may improve ORR for immunotherapy.

## 1. Introduction

Immunotherapy has emerged as a groundbreaking approach to cancer treatment in recent years. Immune checkpoint blockade by targeting the programmed death-ligand 1 (PD-L1)/programmed cell death protein 1 (PD-1) pathway has demonstrated remarkable effectiveness in treating different types of cancer, such as melanoma, non-small cell lung cancer, renal cell carcinoma, and bladder cancer [1,2,3,4,5,6,7]. However, only a small proportion, less than 30%, of patients are responders, though these patients usually have durable clinical responses from targeting the PD-L1/PD-1 pathway [8,9,10]. Studies have shown that PD-L1 expression in tumor tissues is associated with the effectiveness of anti-PD-1/PD-L1 immunotherapy [11,12]. Patients with PD-L1 positive tumors who receive monoclonal antibodies against PD-L1 during immunotherapy can show substantial clinical benefit [13]. Therefore, the precise detection of PD-L1 expression in tumors plays a significant role in guiding clinical treatment decisions.

Conventional methods, such as immunohistochemistry (IHC) and immunofluorescence (IF), for detecting PD-L1 expression have notable limitations [14,15]. These methods depend on tissue sections, thus offering information solely from specific sampled regions and failing to capture the PD-L1 expression profiles throughout the entire tumor. Additionally, they are incapable of capturing dynamic changes in PD-L1 expression and providing limited quantitative measurements. PET is an advanced molecular imaging technology with high sensitivity, target specificity, and in vivo 3D imaging capabilities, which can visualize the target expression status under physiological conditions in real time [16,17,18]. PET imaging agents with high specificity can be synthesized by attaching radiotracers to monoclonal antibodies, peptides, or small molecule inhibitors that specifically target PD-L1. Subsequent PET imaging analysis enables non-invasive, real-time, and quantitative detection of PD-L1 expression in tumor tissues of patients. Recent reports have described PET targeting probes for PD-L1, including antibody-based probes [19,20,21,22,23], peptide-based probes [24,25,26,27], and small molecule inhibitors [28,29,30]. These probes have been utilized to assess the expression status of PD-L1 in patients with various types of solid tumors [19,27,31,32]. However, radiolabeled monoclonal antibodies (mAbs) as molecular probes have a large molecular weight and a long biological half-life, typically necessitating several days to acquire high-contrast images. The use of long half-life radionuclide for labeling mAbs typically results in heightened radiation exposure in patients. Peptide-based imaging agents exhibit lower target specificity, and their molecular properties pose challenges for chemical modifications, thus complicating the regulation of their biodistribution in vivo and pharmacokinetic performance.

**Figure 1:**
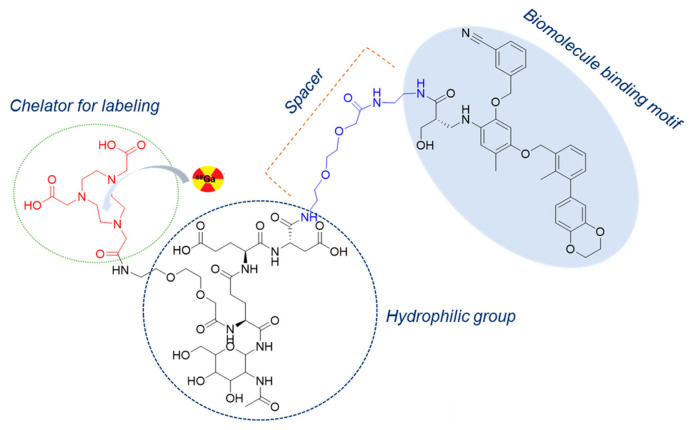
Design and chemical structure of [^68^Ga]BMSH for imaging PD-L1 expression.

With advancements in the study of immune checkpoint molecules, small molecule inhibitors targeting the PD-L1 binding site have been further developed [33,34,35,36]. Small molecule inhibitors offer significant advantages, such as lower molecular weight, ease of modification, better penetration into solid tumors, rapid diffusion in the tumor micro-environment, and uniform distribution in the entire tumor. Lately, several reports have demonstrated that small molecules containing biarylmethyl aryl ether scaffold show high affinity for PD-L1 [28,37,38]. The PET tracer [^18^F]LP-F, derived from small molecule inhibitors that target PD-L1, is capable of effectively identify tumors with varying expression levels within the body [29]. However, its utilization is impeded by the significant lipophilicity of the biarylmethyl aryl ether scaffold. This attribute diminishes the metabolic clearance rate of [^18^F]LP-F, leading to heightened drug accumulation in multiple organs, including the heart, lungs, liver, kidneys, and gastrointestinal tract. In this study, hydrophilic functional groups, biarylmethyl aryl ether scaffold (BMS-1001) linked with 1,4,7-triazacyclononane-1,4,7-triacetic acid (NOTA), were introduced to decrease drug uptake by organs such as the lungs, liver, and gastrointestinal tract (Figure 1), aiming to improve the PD-L1 imaging capability of PET probes derived from small molecule inhibitors. The characteristics of in vitro, biodistributed, and micro-PET image results of the PET radiotracer [^68^Ga]BMSH were investigated.

## 2. Results

### 2.1. Chemical and Radiochemical Properties

**Scheme 1:**
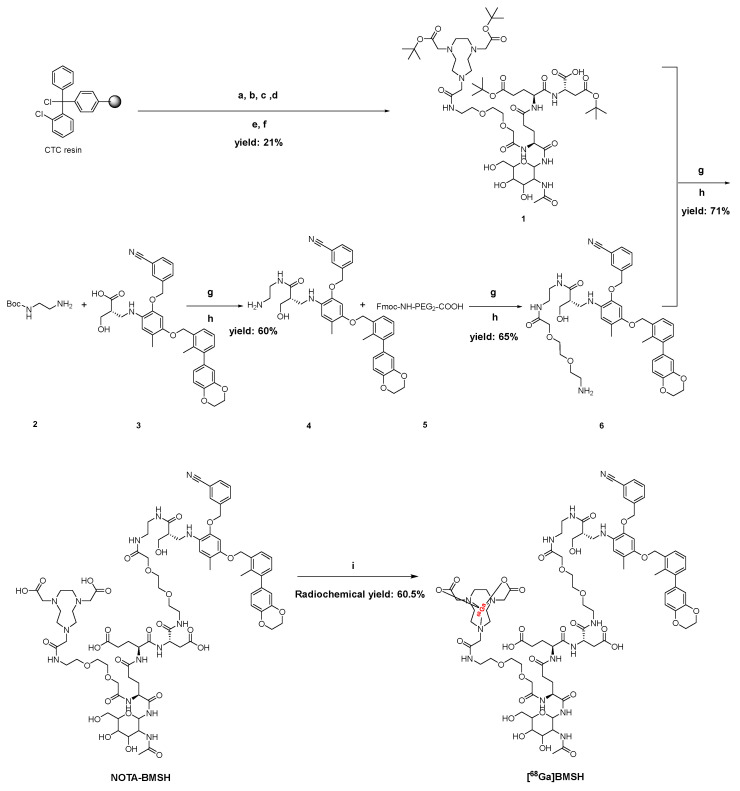
Synthesis of the NOTA-BMSH and [^68^Ga]BMSH, reagents, and conditions: (a) Fmoc-Asp(OtBu)-OH; DIPEA; DCM; 20% piperidine/DMF; (b) Fmoc-Glu(OtBu)-OH; DIC; HOBt; DMF; 20% piperidine/DMF; (c) Fmoc-Glu(2-ACETAMIDO-2-DEOXY-BETA-D-GLUCOSAMINE) -OH; DIC; HOBt; DMF; 20% piperidine/DMF; (d) Fmoc-PEG_2_-OH; DIC; HOBt; DMF; 20% piperidine/DMF; (e) NOTA; DIC; HOBt; DMF; 20% piperidine/DMF; (f) 1% TFA/DCM; (g) HATU; DIPEA DMF; (h) TFA; (i) ^68^GaCl_3_; NaOAc; 55 °C; 10 min.

We designed and synthesized the precursor, NOTA-BMSH, using solid resin and standard Fmoc solid-phase synthesis (Scheme 1). The key intermediate compound **1**, compound **6**, and the labeling precursor were obtained at >95% chemical purity and identified using mass spectrometry (Supplementary Materials, Figures S1–S3). [^68^Ga]BMSH was successfully radiolabeled via complexation of ^68^Ga by NOTA chelator according to Scheme 1. The radioactive product, [^68^Ga]BMSH, was obtained with a non-decay corrected radiochemical yield of 60.5% ± 7.0% (*n* > 10). Its radiochemical purity was >98% with a retention time of 12.6 min when analyzed using radio-HPLC (Figure 1). The specific activity was determined to be 2–8 GBq/μmol (*n* > 10) based on radioactivity measurement. The total time of radiosynthesis was approximately 20 min, including the radiolabeling and purification processes.

### 2.2. Characterization of [^68^Ga]BMSH

**Figure 2:**
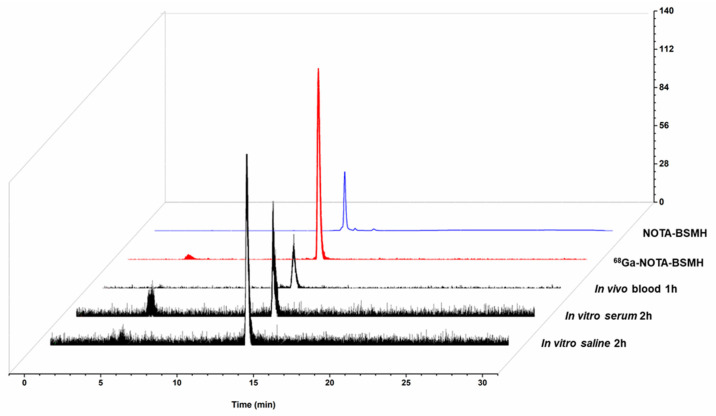
Identification and stability of [^68^Ga]BMSH. HPLC analysis of stability in saline and human serum after incubation at 37 °C for 120 min and in mouse blood at 60 min p.i. The quality control radioactive chromatography of [^68^Ga]BMSH, as well as the UV chromatography of NOTA-BMSH.

The stability of [^68^Ga]BMSH in PBS (37 °C, 2 h), human serum (37 °C, 2 h), and mouse blood (in vivo, 1 h) are shown in Figure 2. In vitro and in vivo stability experiments revealed no significant [^68^Ga]BMSH metabolites were observed on the HPLC chromatogram, indicating the stability of [^68^Ga]BMSH in vivo and in vitro within the testing interval. The log D value of [^68^Ga]BMSH was determined to be −2.02 ± 0.09, indicating its high hydrophilicity.

### 2.3. In Vitro Evaluation of [^68^Ga]BMSH

**Figure 3:**
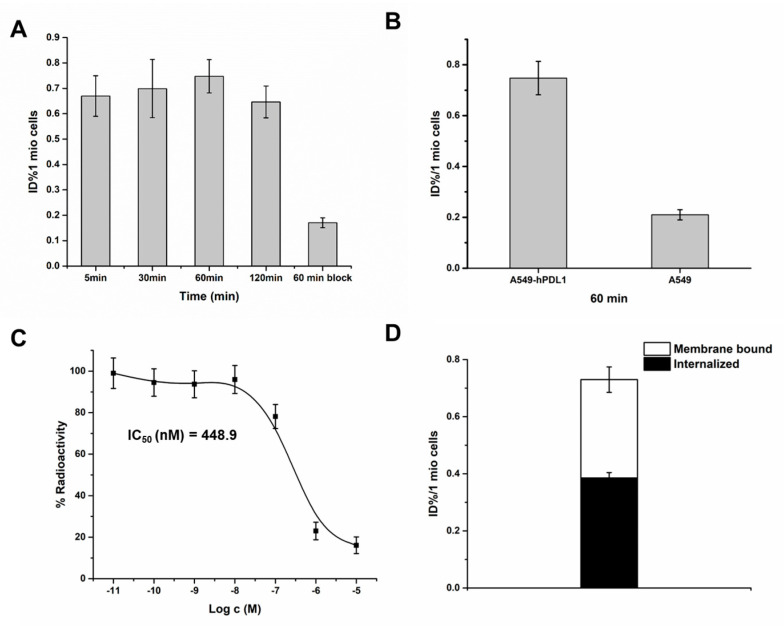
(**A**) In vitro uptake studies of [^68^Ga]BMSH in A549-hPDL1 cells at different time points and blocking studies at 60 min; (**B**) cell uptake on A549-hPDL1 and A549 cells at 60 min; (**C**) competitive binding of NOTA-BMSH against [^68^Ga]BMSH; (**D**) membrane-bound and internalized [^68^Ga]BMSH in A549-hPDL1 cells after incubation for 60 min.

Cell uptake studies were conducted to verify the specific binding ability of [^68^Ga]BMSH to PDL1-expressing tumor cells. The accumulation of [^68^Ga]BMSH in A549-hPDL1 (PDL1-expressing) cells quickly reached 0.67 ± 0.08 ID%/1 mio cells at 5 min, and remained stable at 0.65 ± 0.06 ID%/1 mio cells at 120 min. The maximum uptake value was determined to be 0.74 ± 0.07 ID%/1 mio cells as shown in Figure 3A. After blockade by NOTA-BMSH, the uptake of [^68^Ga]BMSH in A549-hPDL1 cells obviously decreased to 0.17 ± 0.01 ID%/1 mio cells at 60 min. For A549 (PD-L1 negative) cells, the uptake of [^68^Ga]BMSH was at a low level. The uptake value was determined to be 0.21 ± 0.02 ID%/1 mio cells at 60 min, indicating its high specificity for PD-L1 (Figure 3B). The half maximal inhibitory concentration (IC_50_) value of NOTA-BMSH to the A549-hPDL1 cells was measured to be 448.9 nM by competitive cell-binding experiments (Figure 3C). Internalization assays in A549-hPDL1 cells demonstrated a modest uptake of [^68^Ga]BMSH after 60 min incubation with 51.5 ± 2.1% (internalized/total bound activity) (Figure 3D). Efflux experiments demonstrated that [^68^Ga]BMSH exhibited a slightly faster cellular efflux rate in vitro (Figure S4), showing retention of 16.2% of the originally accumulated radioactivity after 180 min, in A549-hPDL1 cells.

### 2.4. Biodistribution of [^68^Ga]BMSH

Receptor-specific uptake was determined using nude mice bearing either PD-L1-positive A549-hPDL1 cells or the negative control tumor, A549. The biodistribution studies of [^68^Ga]BMSH after 30, 60, and 120 min of injection are summarized in Table 1. At 30, 60, and 120 min after injection, the A549-hPDL1 tumor uptake values of the [^68^Ga]BMSH probe were 4.61 ± 0.16, 4.40 ± 0.36, and 4.22 ± 0.65%ID/g, respectively. These observations demonstrated rapid uptake and excellent retention of [^68^Ga]BMSH in the A549-hPDL1 tumor. Although other tissues exhibited relatively high uptake values of the [^68^Ga]BMSH at 30 min post-injection, their uptake notably declined as time elapsed. Using the lung as an illustrative case, the uptake value stood at 8.38 ± 0.54%ID/g at 30 min post-injection, progressively diminishing to 3.01 ± 0.53%ID/g at 120 min post-injection. Meanwhile, the ratio of drug uptake between the tumor and the muscle increased from 3.49 ± 0.21 to 5.78 ± 0.60. In addition, results from the negative control study revealed that the uptake of [^68^Ga]BMSH in A549-hPDL1 tumors was 1.9 times that in A549 tumors (4.22 ± 0.65%ID/g vs. 2.23 ± 0.41% ID/g, at 2 h post-injection, *p* < 0.01), while the biodistribution in the normal organs of A549-hPDL1 and A549 tumor-bearing mice was similar.

### 2.5. Micro-PET Imaging and Immunohistochemical Staining

**Figure 4:**
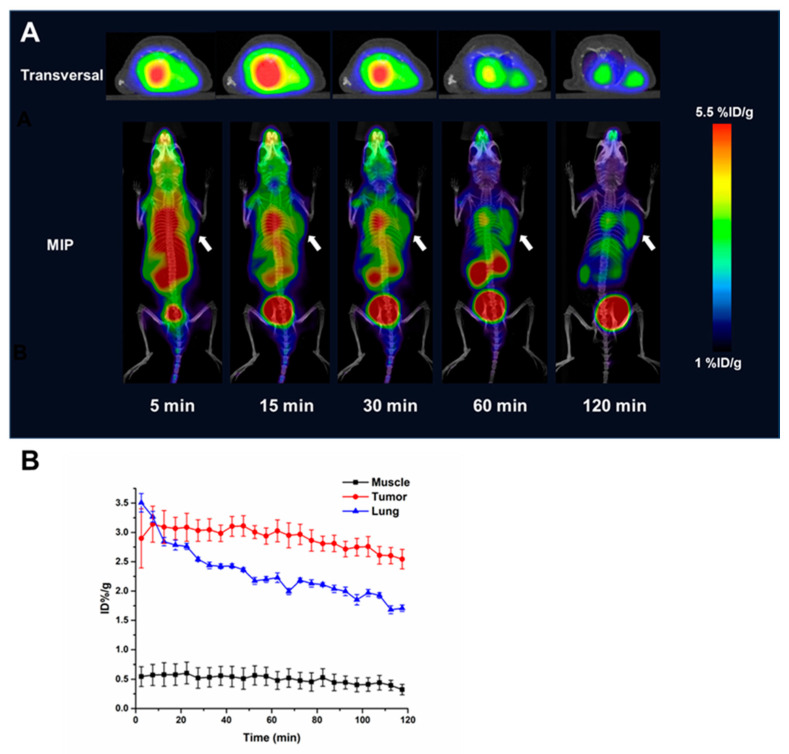
(**A**) The micro-PET/CT images of [^68^Ga]BMSH in A549-hPDL1 tumor-bearing nude mice, where white arrow circle indicates glioma area; (**B**) time–activity curves of [^68^Ga]BMSH uptake in lungs, muscles, and tumors.

To further evaluate the capability of [^68^Ga]BMSH to target PD-L1 in vivo, dynamic micro-PET scans in A549-hPDL1 tumor-bearing mice were performed. Typical micro-PET/CT images (Transversal and MIP images) and time-activity curves of [^68^Ga]BMSH uptake were obtained, as shown in Figure 4. The images demonstrated the rapid uptake of [^68^Ga]BMSH by tumors, lungs, livers, and kidneys, with an extended retention time observed within the tumor region. Simultaneously, there was swift clearance of [^68^Ga]BMSH from the lungs and kidneys, which was consistent with the biodistribution study. Notably, [^68^Ga]BMSH uptake in A549-hPDL1 tumors peaked at 10 min and remained stable until 120 min. At 120 min, the tumor/muscle and the tumor/lung ratio were 7.88 and 1.49, respectively (Figure 4B), whose contrast was high enough for PD-L1 imaging.

**Figure 5:**
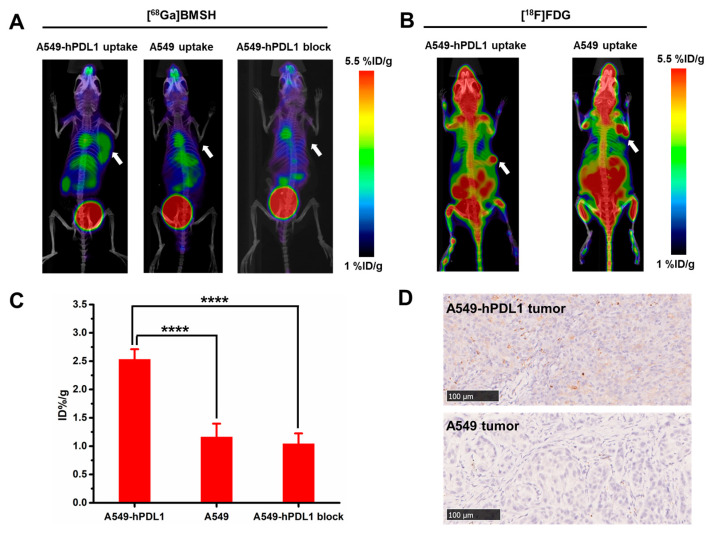
(**A**) Representative MIP images after intravenous injection of [^68^Ga]BMSH at 120 min of A549-hPDL1 xenografts (**A**, **left**) and A549 xenografts (**A**, **middle**), [^68^Ga]BMSH together with the competitor (**A**, **right**), MIP images of [^18^F]FDG in A549-hPDL1 (**B**, **left**) and A549 (**B**, **right**) at 60 min. The tumors are delineated in white arrows; (**C**) uptake or block value of A549-hPDL1, A549 tumor at 120 min (****, *p* < 0.0001); (**D**) IHC staining results of A549-hPDL1 and A549 tumor tissues.

To further verify the diagnosis of PD-L1 expression in vivo, a comparison of tumor uptake and blocking experiments of [^68^Ga]BMSH and [^18^F]FDG were performed with mice bearing A549-hPDL1 or A549 tumors (Figure 5). The 2-h static measurement revealed a notably higher uptake of [^68^Ga]BMSH in A549-hPDL1 tumors compared to A549 tumors (*p* < 0.05) (Figure 5A,C). The blocking study in mice bearing the A549-hPDL1 tumor showed a remarkable decrease in the uptake by the tumor and liver. In contrast, the commonly employed clinical imaging tracer [^18^F]FDG exhibited an elevated uptake in both tumor types, along with a notably high uptake in muscle tissue at 60 min post-injection (Figure 5B, A549-hPDL1 tumor/A549 tumor = 1.08). This underscores the insufficiency of [^18^F]FDG for accurately analyzing PD-L1 expression within the body. The IHC results further revealed that the tumor developed in the A549-hPDL1 tumors had a higher expression of PD-L1 than A549 tumors (Figure 5D).

### 2.6. Internal Dose Assessment

The internal radiation dose was estimated based on the biodistribution of [^68^Ga]BMSH after in vivo injection in nude mice bearing A549-hPDL1 tumor. The organs were estimated to receive low doses of [^68^Ga]BMSH (Table S1). The effective internal radiation dose of [^68^Ga]BMSH was calculated to be 8.74 µSv/MBq for men (Table S1), which was below the single-study FDA limit for research subjects.

## 3. Discussion

Studies have shown that PD-L1 expression in tumor tissues is associated with the effectiveness of anti-PD-1/PD-L1 immunotherapy. The PET tracer [^18^F]LP-F, derived from a biarylmethyl aryl ether scaffold that targets PD-L1, is capable of effectively differentiating tumors with varying expression levels within the body. However, its utilization is impeded by the significant lipophilicity of the biarylmethyl aryl ether scaffold. The carbohydration has been proven to improve general pharmacokinetics, which could significantly increase the kidney clearance and reduce hepatobiliary excretion [39]. Previous studies postulate that introducing a PEG linker can increase the metabolic stability and in vivo half-life of the products [40,41]. The introduction of hydrophilic linkers such as PEG and amino acids such as Asp and Glu to the radiotracer [^68^Ga]BMSH could increase the hydrophilicity and reduce the lipophilicity of [^68^Ga]BMSH, which promotes the rapid renal metabolism of [^68^Ga]BMSH.

In this study, we have reported the successful synthesis as well as the in vitro and in vivo characterization of a new ^68^Ga-labelled PD-L1 tracer and compared it to the clinically used probe, [^18^F]FDG. [^68^Ga]BMSH showed high in vitro and in vivo stability as proved by the radio-HPLC after incubation in PBS, human serum, and mouse blood. The significant difference (*p* < 0.05) of the cellular uptake between the A549-hPDL1 and A549 cells demonstrated that [^68^Ga]BMSH could specifically accumulate in high PD-L1 expression cells. The high uptake of [^68^Ga]BMSH at 5 min in A549-hPDL1 cells demonstrated that [^68^Ga]BMSH could enter cells rapidly. Additionally, the non-radioactive probe NOTA-BMSH could significantly inhibit the uptake of [^68^Ga]BMSH in A549-hPDL1 tumor cells, further indicating the specificity of [^68^Ga]BMSH to PD-L1. The half-inhibitory concentration (IC_50_) of NOTA-BMSH was 448.9 nM, inferior to that of BMS-1001 (2.25 nM), indicating a little weaker affinity of NOTA-BMSH. Differences in affinity exhibited were due to structural changes resulting from the introduction of Asp, Glu, and NOTA. In addition, the relatively high internalization of [^68^Ga]BMSH in A549-hPDL1 cells suggests a potential for extended retention in tumor tissues. Subsequent PET imaging further demonstrated the sustained high uptake of [^68^Ga]BMSH in A549-hPDL1 tumor tissue after 120 min administration.

Ex vivo biodistribution studies of [^68^Ga]BMSH revealed a high uptake (4.22 ± 0.65%ID/g) in PD-L1-positive A549-hPDL1 tumors with a high tumor-to-muscle ratio (5.78 ± 0.60) after 120 min administration. The uptake in non-target tissues was rather low (<2%ID/g), except for in the lung, liver, kidney and blood, which exhibited moderate (3.01 ± 0.53%ID/g), (3.08 ± 0.53%ID/g), (3.94 ± 0.21%ID/g), and high uptake (7.80 ± 1.14), respectively. It is worth highlighting that despite the higher lung uptake observed 30 min post-injection (8.38 ± 0.54%ID/g), there is a notable decrease in lung uptake over time, reaching a value of 3.01 ± 0.53%ID/g at 120 min. This value is even lower than the tumor uptake value of 4.22 ± 0.65%ID/g. According to the difference between [^68^Ga]BMSH in lung and tumor retention time, it is expected to be used in the follow-up clinical translational research for dynamic imaging of lung cancer.

Micro-PET imaging results of A549-hPDL1 and A549 xenografts also demonstrated that [^68^Ga]BMSH can specifically bind to the tumor and remained in the tumor for a longer period of time, which has significant advantages over [^18^F]FDG. Moreover, the accumulation of [^68^Ga]BMSH in A549-hPDL1 tumors could be blocked by co-injection of additional unlabeled inhibitors, indicating that it was a PD-L1-specific uptake. [^68^Ga]BMSH’s internal radiation doses in humans are also deemed safe for clinical application. In short, we clearly demonstrated the feasibility of PET imaging in evaluating PD-L1 expression in xenograft mice tumor models using [^68^Ga]BMSH as a new radiotracer.

## 4. Materials and Methods

### 4.1. Reagents and Instruments

All reagents used in the experiment were commercially purchased and utilized without additional purification, unless specified otherwise. The HPLC system employed was the Agilent 1260 Infinity II (CA, USA), which was equipped with a UV-detector set at 254 nm and C-18 columns. The radiation value of the cell experiment and biological distribution experiment was measured using a γ-counter (Hidex, Turku, Finland). Small animal imaging was conducted using the Madiclab PSA146 PET/CT/FMT instrument (Madic, Linyi, China). Radioactivity was measured utilizing a Capintec CAPRAC-R dose calibrator (NJ, USA).

[^68^Ga]Gallium derived from a prototype 40-mCi ^68^Ge/^68^Ga generator (Tehran, Iran) and [^18^F]FDG were produced from the company of Guangzhou Atomic High-Tech Medical Technology Co., Ltd. (Guangzhou, China) Sep-Pak C18-Light cartridges were purchased from Waters Associates.

### 4.2. General Procedure for the Synthesis of Resin-Bound Compound ***1***

Compound **1** was synthesized using a solid-phase platform. Briefly, 1.00 g of CTC resin (1% divinylbenzene) was contained in a disposable fritted polypropylene column (20 mL). The resin was swollen in dichloromethane (DCM), shaken for 24 h at room temperature, and subsequently drained. Afterwards, the resin was treated with a DCM solution containing a 3-fold excess of Fmoc-Asp(OtBu)-OH and N,N-Diisopropylethylamine (DIPEA), and shaken for 3 h at room temperature. The remaining Fmoc group was removed using a 20% piperidine in N,N-dimethylformamide (DMF) solution, followed by filtration. The resin was then washed successively with DMF (3 × 5 min), MeOH (2 × 5 min), and DCM (1 × 5 min). After the DCM washing, the beads were allowed to air-dry for 15 min. Subsequently, a solution of a 3-fold excess of Fmoc-Glu(OtBu)-OH and N,N′-diisopropylcarbodiimide (DIC) in DMF, with 1-hydroxybenzotriazole (HOBt), was added to the deprotected resin beads and shaken for 1.5 h at room temperature. The excess reagent was drained, followed by a wash with 20% piperidine/DMF, and the beads were then washed according to the standard procedure. Using the same operating procedures, Fmoc-Glu derivatives [Fmoc-Glu(2-ACETAMIDO-2-DEOXY-BETA-D-GLUCOSAMINE) -OH] and Fmoc-NH-PEG_2_-COOH were loaded onto the CTC resin to complete the condensation reaction. The chelator conjugation was performed through adding NOTA, HOBt, and DIC in DMF and allowing the reaction to proceed for 1.5 h. The solution was decanted, and the beads were subjected to the standard washing protocol. Subsequently, the beads were treated with a solution of 1% trifluoroacetic acid (TFA) in DCM to cleave them. The resulting mixture was filtered, and the filtrate was concentrated under vacuum to obtain compound **1**. The LC-MS calculation for compound **1** (C_56_H_97_N_9_O_22_) yielded a theoretical mass of 1248.43, while the actual observed mass was found to be 1249.5 [M+H]^+^.

### 4.3. General Procedure for the Synthesis of Compound ***6***

Compound **2** (Boc-EDA) and compound **3** (BMS1001) were added to a mixture of HATU/DIPEA in DMF for reaction at room temperature, followed by a TFA solution to remove the Boc group and obtain compound **4**. Subsequently, the results were reacted with a solution of compound **5** (Fmoc-NH-PEG_2_-COOH), HATU, and DIPEA in DMF. A solution of DEA in THF was used to remove the Fmoc group to afford compound **6**. LC-MS calcd for C_43_H_51_N_5_O_9_ 781.91; found, 782.9 [M+H]^+^.

### 4.4. Synthesis of NOTA-BMSH

Compound **1** and compound **6** were added to a mixture of 2-(7-Azabenzotriazol-1-yl)-N,N,N′,N′-tetramethyluronium hexafluorophosphate (HATU)/DIPEA in DMF, and tert-butyl was removed to obtain the NOTA-BMSH. LC-MS calcd for C_83_H_114_N_14_O_30_ 1787.89; found, 895.3 [M/2+H]^+^.

The crude product was precipitated from the concentrated solution by the addition of an excess of chilled diethyl ether. The solvent was then removed, and the solid product was triturated three times with diethyl ether. All the crude product was purified by reverse-phase high-performance liquid chromatography (RP-HPLC) carried out on Phenomenex C-18 columns (10 mm × 250 mm, 5 µm), using a linear gradient starting from 90% A (0.1% TFA in water) and 10% B (0.1% TFA in acetonitrile) for 2 min, gradually decreasing to 20% A at 15 min at a flow rate of 3 mL/min.

### 4.5. Radiochemical Synthesis of [^68^Ga]BMSH

The ^68^Ga labeling process involved elution of a ^68^Ge/^68^Ga generator in fractions using 4 mL of 0.25 M HCl. The NOTA-BMSH aqueous solution (50 µg in 50 µL of deionized water) was then buffered with 0.25 M sodium acetate (1 mL), followed by the addition of 4 mL of ^68^Ga hydrochloric acid solution. Subsequently, the mixture was incubated at 55 °C for 10 min to fully complete the radiolabeling reaction. After the reaction, the mixture was cooled in an ice bath and diluted with 5 mL of water. The diluted mixture was loaded onto an activated C18 cartridge and underwent sequential washing with 30 mL of water. The desired radiolabeled compound was eluted using a 1 mL mixture of ethanol and water (1:1, *v*/*v*). The eluent was then filtered through a sterile 0.2 μm filter membrane and further diluted with saline for subsequent studies. The radiochemical purity of [^68^Ga]BMSH was assessed by utilizing an analytical HPLC column (Phenomenex C18) under the aforementioned analytic conditions.

### 4.6. Partition Coefficient

[^68^Ga]BMSH (37 kBq) was added to a mixture comprising 5.0 mL of phosphate-buffered saline (PBS) with a pH of 7.4 and 5.0 mL of 1-octanol in a 15 mL centrifuge tube. The resulting mixture underwent vigorous vortexing for 5 min and was subsequently centrifuged at 10,000 rpm for 5 min. Samples of 100 µL were extracted from each phase, and the radioactivity was quantified using a γ-counter. The partition coefficient was calculated as Log_10_D = Log_10_ (counts in 1-octanol/counts in PBS) (*n* = 3).

### 4.7. In Vitro Serum Stability and In Vivo Stability

[^68^Ga]BMSH (3.7 MBq) was added into normal human serum (0.2 mL) and incubated for 2 h at 37 °C. Plasma protein was precipitated with 0.4 mL acetonitrile and centrifuged (10,000 rpm, 5 min). The radiochemical purities of [^68^Ga]BMSH in filtrates were assayed by analytic HPLC under analytic conditions as described above.

BALB/c-Nude mice were injected intravenously with [^68^Ga]BMSH (0.185 GBq/kg). The mice were sacrificed 60 min after injection, and the mouse blood samples (0.4 mL) were collected. An equal volume of acetonitrile was added to the blood and centrifuged at 10,000 rpm for 5 min; the supernatant was filtered through a 0.45-mm syringe filter. The blood supernatant sample (200 µL) was then injected onto analytic HPLC under analytic conditions as described above.

### 4.8. Cell Lines and Tumor Models

Stably PD-L1-transfected A549-hPDL1 cells (acquired using lentiviral infection) and A549 cells (purchased from the Institute of Biochemistry and Cell Biology, Shanghai, China) were used for the cell-based experiments. A549-hPDL1 cells and A549 cells were cultured in RPMI-1640 medium (Gibco, Carlsbad, CA, USA); both medium were supplemented with 15% fetal bovine serum (Gibco) and antibiotics (100 mg/mL streptomycin and 100 mg/mL penicillin; Gibco) at 37 °C in a humidified incubator with 5% CO_2_.

For biodistribution and micro-PET imaging studies of [^68^Ga]BMSH and [^18^F]FDG, male BALB/c nude mice were implanted subcutaneously with 1–5 × 10^6^ A549-hPDL1 cells and A549 cells behind right armpit. Mice were imaged or used in biodistribution studies when the tumor xenografts reached 5–10 mm in diameter.

### 4.9. In Vitro Cell Study

A549-hPDL1 cells and A549 cells were grown in flasks to 80–90% confluence and were used for cell uptake studies. Sets of four 12-well plates containing 1 × 10^5^ cells/well were incubated with [^68^Ga]BMSH (1.85 kBq) for 5, 30, 60, and 120 min at 37 °C. After removing the medium, the cells were washed twice with ice-cold PBS, pH 7.4. The adherent cells were then washed twice with glycine and lysed with 1 N NaOH at 37 °C for 10 min. The combined washes and lysate were measured with a γ-counter.

Subsequently, the blocking study was conducted in A549-hPDL1 and A549 cells. The cells were incubated for 60 min at 37 °C with [^68^Ga]BMSH (1.85 kBq), with or without the pretreatment of NOTA-BMSH, (10 µg/well). After removing the medium, the cells were washed twice with ice-cold PBS. The cells were then washed twice with glycine and lysed with 1 M NaOH at 37 °C for 10 min. The combined washes and lysate were measured with a γ-counter. The result was expressed as %ID/1 mio cells.

Half-inhibitory concentration (IC_50_) of NOTA-BMSH to A549-hPDL1 cells were measured by simultaneous exposure to unlabeled (10^−5^ M to 10^−11^ M) and radiolabeled compound for 60 min.

### 4.10. Tissue Biodistribution Studies

The mice (A549-hPDL1 and A549, *n* = 3 in each group) were euthanized 0.5 h, 1 h, 2 h pi of [^68^Ga]BMSH (148–222 kBq), the organs and tumors were removed, weighed, and radioactivity was counted using a γ-counter. The radioactivity in each organ was normalized as the percentage of injected dose per gram of tissue (%ID/g).

### 4.11. Micro-PET Scanner Imaging

Dynamic micro-PET imaging studies were conducted in tumor-bearing nude mice (A549-hPDL1 and A549, *n* = 3 in each group) using the Madiclab PSA146 PET/CT/FMT instrument (Linyi, China). The images were reconstructed using a three-dimensional ordered-subset expectation maximum (OSEM) algorithm and were converted to the percentage of injected dose per gram of tissue (%ID/g) images. Tumor-bearing mice were intravenously injected with [^68^Ga]BMSH (7.4 MBq/mouse) and [^18^F]FDG (5.55 MBq/mouse), respectively. For the blocking experiment, the A549-hPDL1 tumor-bearing mice were co-injected with NOTA-BMSH (100 µg/mouse) and [^68^Ga]BMSH. For data analysis, the regions of interest (ROIs) were manually drawn over the tumor and major organs on decay-corrected whole-body coronal images using PMOD software (version 4.3, PMOD Technologies Ltd., Zurich, Switzerland).

### 4.12. Immunohistochemical Staining

Tumor tissues of A549-hPDL1 and A549 tumor-bearing mice obtained by sacrificing the mice were immunohistochemically stained after preparation of paraffin-embedded sections using 4% paraformaldehyde. The sections were incubated with the PDL1-alpha primary antibody (diluted 1:200, Affinity) and then with the goat anti-mouse secondary antibody (diluted 1:200, Servicebio). The secondary antibody was a molecule formed by combining horseradish peroxidase (HRP) and goat anti-mouse IgG. The sections were then reacted with DAB staining solution after combining the primary and secondary antibodies. DAB produced brown precipitation upon HRP catalysis, which amplified the signal and developed color. The immunohistochemical images were finally obtained after a series of routine processing.

### 4.13. Internal Radiation Dose of [^68^Ga]BMSH

Internal radiation dose was estimated based on the biodistribution of [^68^Ga]BMSH for in vivo injection nude female mice bearing A549–hPDL1 tumor. The radiation dose estimates were calculated for human organs, based on an extrapolation of the animal data to humans using OLINDA (v.1.0 (2003)/EXM software.

### 4.14. Statistical Analysis

Quantitative data were reported as mean ± standard deviation (SD), and statistical differences between groups were assessed using Student’s *t*-test conducted with Origin 9.1 software. A *p*-value of less than 0.05 was considered statistically significant.

## 5. Conclusions

In conclusion, the investigated small molecule peptide compound, [^68^Ga]BMSH, labeled with ^68^Ga, underwent comprehensive in vitro and in vivo evaluations, demonstrating promising outcomes for tumor imaging of PD-L1 expression in murine subjects. In comparison to [^18^F]FDG, [^68^Ga]BMSH exhibited the ability to selectively identify tumors with varying levels of PD-L1 expression. In summary, these findings underscore the substantial potential of [^68^Ga]BMSH in detecting PD-L1 expression and suggest further prospects for its clinical translation in the future.

## Figures and Tables

**Table 1:** Biodistribution of [^68^Ga]BMSH in different mice organs at 30, 60, and 120 min pi. Values are expressed as means ± SD (% ID/g) (*n* = 3).

Organ	30 min	60 min	120 min
Bone	1.60 ± 0.33	1.38 ± 0.20	0.88 ± 0.19
Muscle	1.39 ± 0.13	1.09 ± 0.12	0.73 ± 0.27
Lung	8.38 ± 0.54	7.16 ± 1.06	3.01 ± 0.53
Brain	0.32 ± 0.03	0.25 ± 0.02	0.19 ± 0.02
Heart	3.70 ± 0.08	3.10 ± 0.47	2.00 ± 0.33
Liver	3.73 ± 0.25	3.76 ± 0.22	3.08 ± 0.35
Kidney	6.42 ± 1.34	6.12 ± 0.23	3.94± 0.21
Spleen	2.37 ± 0.18	1.84 ± 0.05	1.31 ± 0.28
Gall bladder	1.97 ± 0.96	1.10 ± 0.18	1.33 ± 0.43
Stomach	2.86 ± 0.83	2.30 ± 0.51	1.26 ± 0.10
Intestine	1.71 ± 0.03	1.66 ± 0.33	1.71 ± 0.36
Blood	16.21 ±3.02	11.49 ± 1.67	7.80 ± 1.14
A549-hPDL1 tumor	4.61 ± 0.16	4.40 ± 0.36	4.22 ± 0.65
A549 tumor	-	-	2.23 ± 0.41
A549-hPDL1 T/M	3.49 ± 0.21	4.32 ± 0.84	5.78 ± 0.60
A549-hPDL1 T/L	0.58 ± 0.05	0.65 ± 0.07	1.26 ± 0.03

## Data Availability

Data are contained within the article or Supplementary Materials.

## Supplementary Materials

The following supporting information can be downloaded at: https://www.mdpi.com/article/10.3390/ph16101487/s1, Figure S1: ESI-MS spectrum of compound **1**; Figure S2: ESI-MS spectrum of compound **6**; Figure S3: ESI-MS spectrum of NOTA-BMSH; Figure S4: efflux kinetics of [^68^Ga]BMSH after incubation of A549-hPDL1 cells with radiolabeled compounds for 60 min followed by incubation with a compound–free medium for 0–180 min; Table S1: estimated human dosimetry data of [^68^Ga]BMSH in mSv/MBq (male).

## Abbreviations

ACN, Acetonitrile; DMF, N,N-dimethylformamide; DIPEA, N,N-diisopropylethylamine; PBS, phosphate–buffered saline; DIC, N,N’–diisopropyl carbodiimide; HOBt, 1–hydroxybenzotriazole; TFA, trifluoroacetic acid; DCM, dichloromethane; HPLC, high performance liquid chromatography; MIP, maximum density projection; PET, positron emission tomography.
