# Supplementary material for: Development and Preclinical Evaluation of [68Ga]BMSH as a New Potent Positron Emission Tomography Tracer for Imaging Programmed Death-Ligand 1 Expression
Source: Pharmaceuticals (Basel). 2023 Oct 19;16(10):1487. doi: 10.3390/ph16101487 (PMC10610256; doi:10.3390/ph16101487)
Supplement: Supplementary file 1 [file pharmaceuticals-16-01487-s001.zip › pharmaceuticals-2646176-supplementary.pdf]

Supplemental Information

Article

Development and Preclinical Evaluation of [^{68}Ga]BMSH as a New Potent Positron Emission Tomography Tracer for Imaging Programmed Death-Ligand 1 Expression

Yong Huang [1,†], Chengze Li [1,†], Zhongjing Li [1], Qiong Wang [1], Size Huang [1], Qi Liu [2,3,*] and Ying Liang [1,*]

[1] Department of Nuclear Medicine, National Cancer Center, National Clinical Research Center for Cancer, Cancer Hospital & Shenzhen Hospital, Chinese Academy of Medical Sciences and Peking Union Medical College, Shenzhen 518116, China

[2] International Cancer Center, Shenzhen University School of Medicine, Shenzhen University, Shenzhen 518057, China

[3] Institute of Biomedical Engineering, Shenzhen Graduate School, Peking University, Shenzhen 518055, China

* Correspondence: liu_qi@szu.edu.cn (Q.L.); liangy_2000@sina.com (Y.L.)

† These authors contributed equally to this work.

1. Characterization.

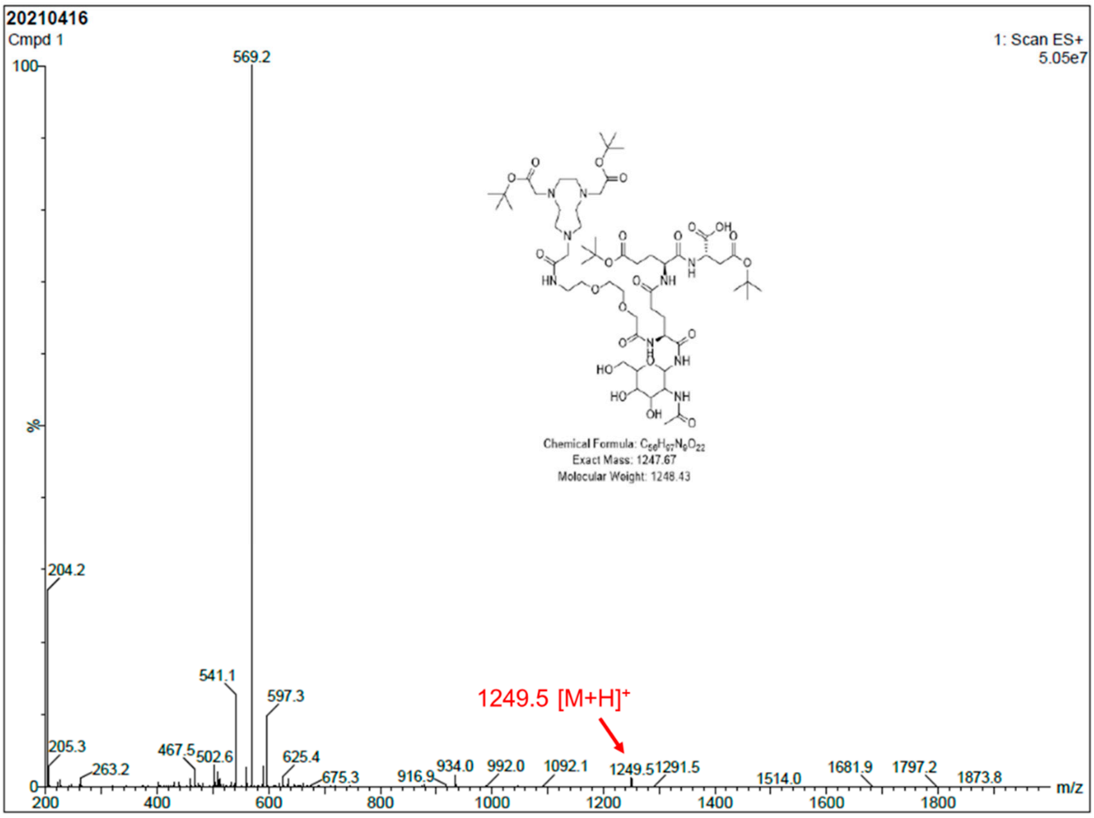

Figure S1. ESI−MS spectrum of compound **1**.

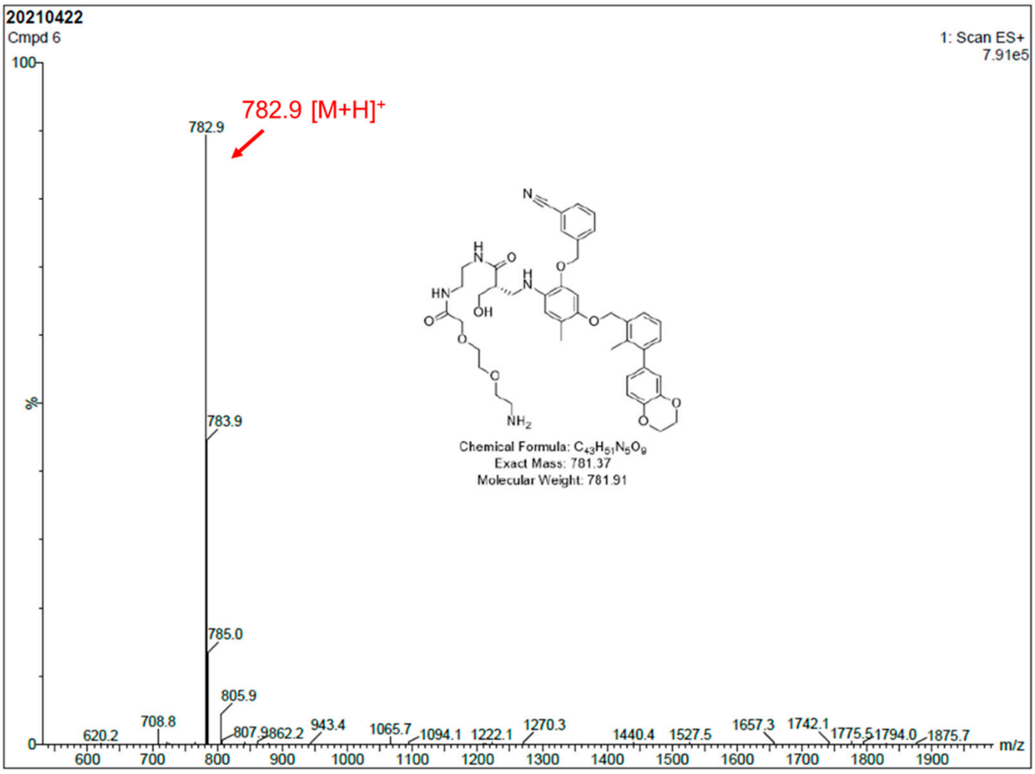

Figure S2. ESI−MS spectrum of compound **6**.

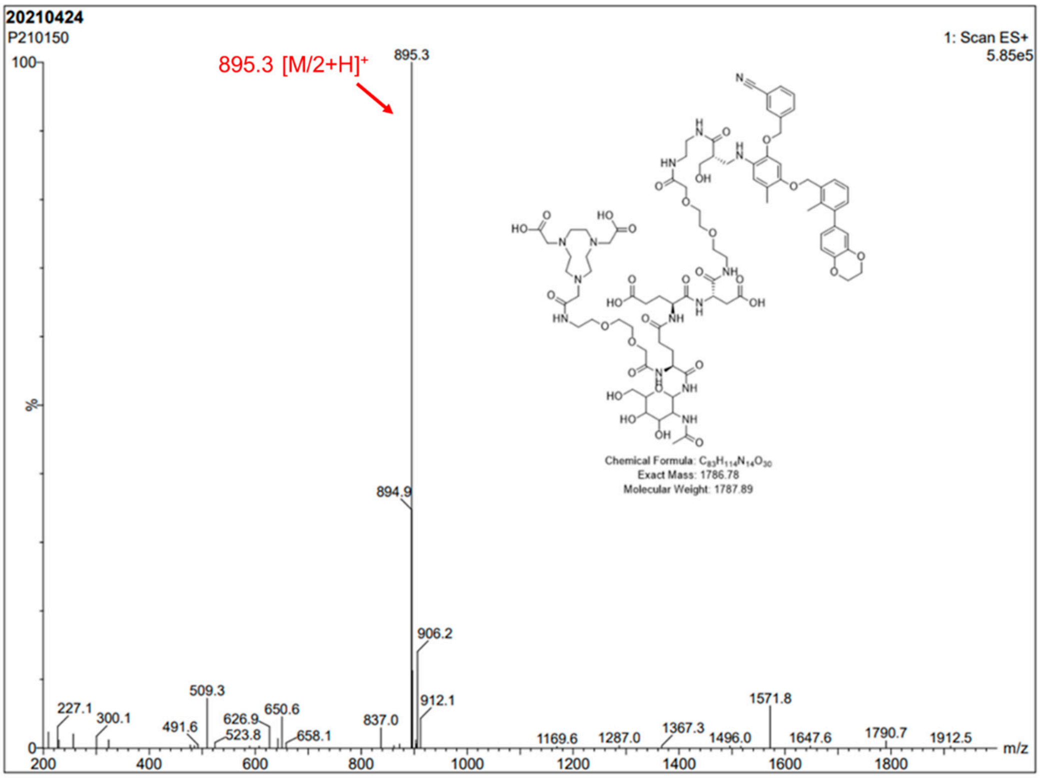

Figure S3. ESI−MS spectrum of NOTA−BMSH

2. Cell study

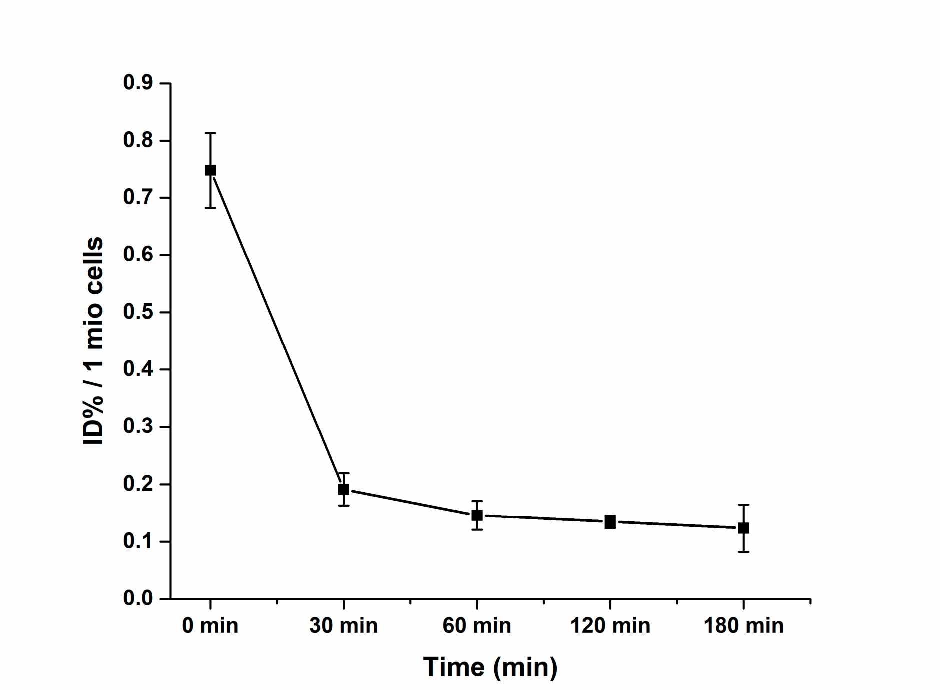

Figure S4. Efflux kinetics of [^{68}Ga]BMSH after incubation of A549−hPDL1 cells with radiolabeled compounds for 60 min followed by incubation with a compound−free medium for 0−180 min.

3. Estimated human dosimetry of [^{68}Ga]BMSH.

Table S1. Estimated human dosimetry data of [^{68}Ga]BMSH in mSv/MBq(male).

Target Organ	Total	EDE Cont.	ED Cont.
Adrenals	1.36E−02	0.00E00	1.26E−04
Brain	1.48E−03	0.00E00	1.48E−05
Esophagus	3.34E−03	0.00E00	1.34E−04
Eyes	1.97E−04	0.00E00	0.00E00
Gallbladder Wall	3.65E−03	0.00E00	3.37E−05
Left colon	2.39E−03	0.00E00	1.16E−04
Small Intestine	1.93E−02	1.16E−03	1.78E−04
Stomach Wall	3.37E−03	0.00E00	4.05E−04
Right colon	1.70E−03	0.00E00	8.22E−05
Rectum	6.02E−04	0.00E00	1.39E−05
Heart Wall	2.53E−02	1.52E−03	2.33E−04
Kidneys	1.22E−01	7.31E−03	1.12E−03
Liver	1.75E−02	1.05E−03	6.99E−04
Lungs	3.52E−02	4.23E−03	4.23E−03
Pancreas	2.97E−03	0.00E00	2.74E−05
Prostate	5.35E−04	0.00E00	2.47E-06
Salivary Glands	2.74E−04	0.00E00	2.74E−06
Red Marrow	3.43E−03	4.12E−04	4.12E−04
Osteogenic Cells	3.26E−03	9.79E−05	3.26E−05
Spleen	8.45E−02	5.07E−03	7.80E−04
Testes	7.35E−05	1.84E−05	2.94E−06
Thymus	3.03E−03	0.00E00	2.79E−05
Thyroid	1.39E−03	4.17E−05	5.55E−05
Urinary Bladder Wall	2.92E−04	0.00E00	1.17E−05
Total Body	2.71E−03	0.00E00	0.00E00
Effective Dose		2.09E−02	8.74E−03
